# Inactivation of *Fam20C* in Cells Expressing Type I Collagen Causes Periodontal Disease in Mice

**DOI:** 10.1371/journal.pone.0114396

**Published:** 2014-12-05

**Authors:** Peihong Liu, Hua Zhang, Chao Liu, Xiaofang Wang, Li Chen, Chunlin Qin

**Affiliations:** 1 Department of Periodontics, Harbin Medical University School of Stomatology, Harbin, Heilongjiang, 150001, China; 2 Department of Biomedical Sciences and Center for Craniofacial Research and Diagnosis, Texas A&M University Baylor College of Dentistry, Dallas, Texas, 75246, United States of America; 3 Longjiang Scholar Laboratory, The First Affiliated Hospital of Harbin Medical University, Harbin, Heilongjiang, 150001, China; University of Southern California, United States of America

## Abstract

**Background:**

FAM20C is a kinase that phosphorylates secretory proteins. Previous studies have shown that FAM20C plays an essential role in the formation and mineralization of bone, dentin and enamel. The present study analyzed the loss-of-function effects of FAM20C on the health of mouse periodontal tissues.

**Methods:**

By crossbreeding *2.3 kb Col 1a1-Cre* mice *with Fam20C^fl/fl^* mice, we created *2.3 kb Col 1a1-Cre;Fam20C^fl/fl^* (cKO) mice, in which *Fam20C* was inactivated in the cells that express Type I collagen. We analyzed the periodontal tissues in the cKO mice using X-ray radiography, histology, scanning electron microscopy and immunohistochemistry approaches.

**Results:**

The cKO mice underwent a remarkable loss of alveolar bone and cementum, along with inflammation of the periodontal ligament and formation of periodontal pockets. The osteocytes and lacuno-canalicular networks in the alveolar bone of the cKO mice showed dramatic abnormalities. The levels of bone sialoprotein, osteopontin, dentin matrix protein 1 and dentin sialoprotein were reduced in the *Fam20C*-deficient alveolar bone and/or cementum, while periostin and fibrillin-1 were decreased in the periodontal ligament of the cKO mice.

**Conclusion:**

Loss of *Fam20C* function leads to periodontal disease in mice. The reduced levels of bone sialoprotein, osteopontin, dentin matrix protein 1, dentin sialoprotein, periostin and fibrillin-1 may contribute to the periodontal defects in the *Fam20C*-deficient mice.

## Introduction

FAM20C is a member of the “family with sequence similarity 20”; in mammals, this evolutionarily conserved protein family consists of three members: FAM20A, FAM20B and FAM20C [1]. Inactivating mutations in the human *FAM20C* gene cause Raine syndrome, an autosomal recessive disorder that demonstrates heterogeneous manifestations [2]–[7]. Patients with the lethal Raine syndrome may die shortly after birth [2], [3], while the nonlethal cases manifesting bone sclerosis and/or hypophosphatemic rickets/osteomalacia may live into adulthood [4], [6], [7].

FAM20C is expressed at significant levels in the mineralized tissues and a number of soft tissues including dentin, enamel, bone, cementum, periodontal ligament (PDL), cerebrum cortex, basal ganglia, skeletal cartilage, heart, liver and kidney [1], [8], [9]. Previously, our group showed that global inactivation of *Fam20C* in mice led to hypophosphatemic rickets, along with a downregulation of certain osteoblast differentiation markers, an elevation of fibroblast growth factor 23 in the serum, and a reduction of serum phosphorus [10]. These *Fam20C*-deficient mice also showed remarkable enamel and dentin defects [11].

*In vitro* studies have shown that FAM20C is a Golgi kinase that phosphorylates serine residues in the Ser-X-Glu (S-X-E) motifs of secretory proteins [12], [13]. The “Small-Integrin-Binding LIgand, N-linked Glycoproteins” (SIBLING) family includes bone sialoprotein (BSP), osteopontin (OPN), dentin matrix protein 1 (DMP1) and dentin sialophosphoprotein (DSPP) [14]. The SIBLING proteins, which are secreted into the extracellular matrix (ECM) of certain mineralized and non-mineralized tissues [15], play important roles in the formation and maintenance of a healthy periodontium [16]–[19]. One of the common features shared by the SIBLING family members is the presence of S-X-E motifs in their amino acid sequences; the serine residues in these motifs are often phosphorylated [14], [15]. *In vitro* studies showed that FAM20C phosphorylates serine residues in the S-X-E motifs of OPN and DMP1 [12], [13], [20]. Periostin and fibrillin-1, two ECM proteins highly expressed in the periodontal ligament [21]–[23], are essential to the health of periodontal tissues [24]–[28]. Periostin and fibrillin-1 have several S-X-E motifs in their amino acid sequences [29]–[31] and, thus, both are potential substrates of FAM20C.

In this study, by crossbreeding the *Fam20C* ^floxed/floxed^ (*Fam20C^fl/fl^*) mice [10] with transgenic mice expressing Cre-recombinase driven by the *2.3 kb Col 1a1* promoter, we generated *2.3 kb Col 1a1-Cre;Fam20C^fl/fl^* (cKO) mice, in which *Fam20C* was inactivated in the cells expressing Type I collagen. We analyzed the periodontal tissues in the cKO mice using X-ray radiography, histology and scanning electron microscopy approaches. We performed immunostaining for BSP, OPN, DMP1, dentin sialoprotein (DSP, the NH_2_-terminal fragment of DSPP), periostin and fibrillin-1 to examine if the levels and distribution of these potential substrates of FAM20C were altered in the *Fam20C*-deficient periodontium. We observed that the *Fam20C*-deficient mice developed periodontal diseases, along with reduced levels of the above secretory proteins in the periodontium.

## Materials and Methods

### Ethics statement

The use of animals in this study was approved by the Institutional Animal Care and Use Committee (IACUC) of Texas A&M University Baylor College of Dentistry (approved protocol numbers: 2011-09-BCD and 2012-03-BCD) and was in strict accordance with the recommendations in the Guide for Care and Use of Laboratory Animals of the National Institutes of Health.

### Generation *of 2.3 kb Col 1a1-Cre;Fam20C^fl/fl^* mice

We first crossbred the *Fam20C^fl/fl^* mice [10] with *2.3 kb Col 1a1-Cre* transgenic mice (a gift from Dr. Jian Feng, Texas A&M University Baylor College of Dentistry, Dallas, Texas, USA) to create *2.3 kb Col 1a1-Cre;Fam20C^fl/+^* mice. The *2.3 kb Col 1a1-Cre;Fam20C^fl/+^* mice were further bred with *Fam20C^fl/fl^* mice to generate *2.3 kb Col 1a1-Cre;Fam20C^fl/fl^* mice, which we refer to as “conditional knockout” (cKO) mice in this report. The *Fam20C^fl/+^* or *Fam20C^fl/fl^* mice from the same litters created during the crossbreeding regime were used as normal controls. Previous studies in our group [10], [11] have shown that *Fam20C^fl/fl^* mice or mice losing one allele of *Fam20C* (i.e., heterozygous *Fam20C* knockout mice) are normal. In this investigation, we also observed that *2.3 kb Col 1a1-Cre;Fam20C^fl/+^* mice were not different from the wild type mice. Using the *Fam20C^fl/+^* or *Fam20C^fl/fl^* littermates of *2.3 kb Col 1a1-Cre;Fam20C^fl/fl^* (cKO) mice as normal controls not only reduced the number of mice needed but also prevented potential variances that may result from comparing mice from different litters. DNA samples isolated from mouse tails were analyzed by polymerase chain reaction (PCR) genotyping with primers specific for the *Cre* transgene and *Fam20C* floxed allele, as we previously described [10], [11].

We observed that the periodontal ligament of the 4-week-old cKO mice did not have significant inflammation and the junctional epithelium in their molars was at normal position. Thus, we selected the 4-week-old mice as the starting point of observation, and chose the 12- and 24-week-old mice to evaluate the progression of periodontal defects in the cKO mice. Samples from the normal mice at the same ages were used as controls in this study. Four to seven mice were analyzed for each age group of the cKO or normal mice. The study was performed in accordance with the Guidelines laid down by the National Institutes of Health in the USA regarding the care and use of animals for experimental procedures. The animal protocol was approved by the Animal Welfare Committee of Texas A&M University Baylor College of Dentistry.

### Plain X-ray radiography and micro-computed tomography (μCT)

The mandibles dissected from the normal and cKO mice at the ages of 4, 12 and 24 weeks were analyzed using plain X-ray radiography (Faxitron MX-20DC12 system; Faxitron Bioptics, Tucson, Arizona, USA). While we also used X-ray radiography to assess the long bone of the cKO mice at the above ages, this report focuses on the loss-of-function effects of *Fam20C* on the periodontal tissues in the mandible. The mandibles dissected from these mice were examined by μCT radiography (Scanco μCT35 imaging system; Scanco Medical, Brüttisellen, Switzerland) using a low-resolution scan (12-µm slice increment) for morphological observations, as previously reported [10], [11]. The data acquired from the high-resolution scans (6-µm slice increment) of the samples from 4 mice (n = 4) at 12 and 24 weeks were used for quantitative analyses. The quantitative data were reported as mean ± SD and analyzed by Student's *t* test. P<0.05 was considered statistically significant in the quantitative analyses.

### Resin-casted scanning electron microscopy (SEM)

For the SEM analyses, the mandibles from 4-week-old mice were dissected and fixed with 4% paraformaldehyde in 0.1 M cacodylate buffer solution (pH 7.4) at 4°C for 24 hours. The tissue specimens were dehydrated in ascending concentrations of ethanol and then embedded in methyl methacrylate (MMA, Buehler, Lake Bluff, Illinois, USA). After adjusting a suitable comparable position of the samples, sandpaper was used to grind the acrylic block in an increasing order of grit fineness. These samples were then polished using a micro cloth with Metadi Supreme Polycrystalline diamond suspension of 0.1, 0.25 and 0.05 microns in size (Buehler). These samples were then washed ultrasonically and placed in the vacuum system for 2 days. To assess the osteocyte and lacuno-canalicular structures, the surface of the MMA-embedded mandible was polished, acid-etched with 12% phosphoric acid for 7 seconds, washed with 5% sodium hypochlorite for 35 minutes, coated with gold and palladium, and then examined using a FEI/Philips XL30 Field emission environmental SEM system (JSM-6010LA, JEOL, Tokyo, Japan).

### Preparation of decalcified sections and haematoxylin and eosin (H&E) staining

The mandibles from 4-, 12- and 24-week-old mice were fixed overnight at 4°C with 4% paraformaldehyde in phosphate buffered saline (PBS) solution and then decalcified in 15% ethylenediaminetetraacetate (EDTA) solution (pH 7.4) at 4°C for 5∼14 days, depending on the ages of the animals. The samples were processed for paraffin embedding, and 5-µm serial sections were prepared for H&E staining, picro-sirius red staining, and immunohistochemistry analyses.

### Picro-sirius red staining

For picro-sirius red staining, the sections were immersed in haematoxylin solution for 8 minutes to stain the nuclei and washed for 10 minutes in water. The sections were then stained in picro-sirius red for one hour, washed in two changes of acidified water, dehydrated in three changes of 100% ethanol, cleared in xylene and mounted. We analyzed the structure and organization of collagen fibers in the periodontal ligament under bright-field and polarized light microscopy.

### Immunohistochemistry (IHC) Staining

The IHC experiments on paraffin-embedded sections were carried out using ABC kit and DAB kit (Vector Laboratories, Burlingame, California, USA) according to the manufacturer's instructions. The polyclonal anti-FAM20C antibody [9] was diluted at 1∶400 and used to analyze the presence or absence of FAM20C in the mandible sections from the normal or *Fam20C*-cKO mice. We employed polyclonal antibodies against BSP (LF87, a gift from Dr. Larry Fisher of the National Institute of Dental and Craniofacial Research), OPN [32], DMP1 [33], and a monoclonal antibody against DSP [34] to detect the individual members of the SIBLING family as we previously reported [34]–[36]. An affinity-purified polyclonal antibody against periostin at a concentration of 1 µg/ml (Innovative Research, Atlanta, Georgia, USA) and an affinity-purified polyclonal antibody at a concentration of 20 µg/ml against fibrillin-1 (Sigma-Aldrich, St. Louis, Missouri, USA) were employed to detect these two ECM molecules in the periodontal tissues according to the manufacturers' instructions. In the IHC analyses for each type of antibodies, the specimens from the normal and cKO mice from the same litters were stained in the same batch of experiments to ensure that exactly the same conditions were applied to the normal and cKO groups. The same concentrations of normal rabbit serum or rabbit IgG were used to replace the polyclonal antibodies serving as negative controls for the IHC experiments detecting BSP, OPN, DMP1, periostin and fibrillin-1. The same concentration of mouse IgG was used to replace the anti-DSP antibody, functioning as a negative control for this monoclonal antibody. The IHC sections were counterstained with methyl green.

## Results

### X-ray radiography

X-ray radiography revealed that the cKO mice have defects in the periodontium, dentin abnormalities in the teeth and a rachitic appearance in the skeleton. The cKO mice also had a smaller stature and a lower level of serum phosphorus compared to the normal mice (data not shown); the serum phosphorus level in the 12-week-old cKO mice was reduced by approximately 50%, similar to that in the mice in which *Fam20C* was globally inactivated (10). This report focuses on the periodontal defects associated with the inactivation of *Fam20C*.

**Figure 1:**
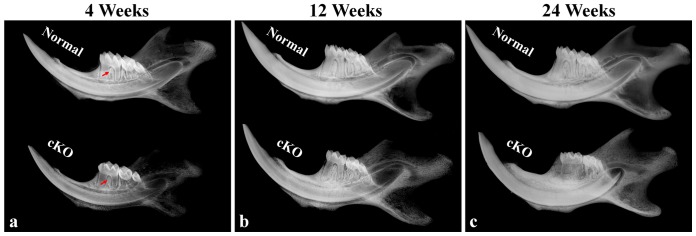
Plain X-ray radiography analyses of 4-, 12- and 24-week-old mice. The mandibles dissected from the 4-, 12- and 24-week-old normal mice (images in the upper portion) and cKO mice (lower portion) were examined by X-ray radiography. At 4 weeks, the furcation region between the mesial and distal roots of the first mandibular molars in the cKO mice had apparent radiolucency compared with the same area of the normal mice (a, arrows). At 12 weeks, the interdental region between the first and second mandibular molars in the cKO mice revealed remarkable bone loss compared with the normal mice (b). At 24 weeks, alveolar bone in the furcation and interdental regions of the cKO mice showed dramatically lower radiopacity than the normal mice (c).

At 4 weeks after birth, plain X-ray examinations showed radiolucency in the furcation region between the first and second mandibular molars of the cKO mice, while the height of the interdental alveolar bone appeared similar in the normal (control) and cKO mice (Figure 1a). At 12 weeks, the interdental region between the first and second mandibular molars in the cKO mice had remarkable bone loss compared with the normal mice (Figure 1b). At 24 weeks, very little alveolar bone remained in the interdental region, and the remaining alveolar bone in the cKO mice had a much lower radiopacity than the normal mice (Figure 1c).

**Figure 2:**
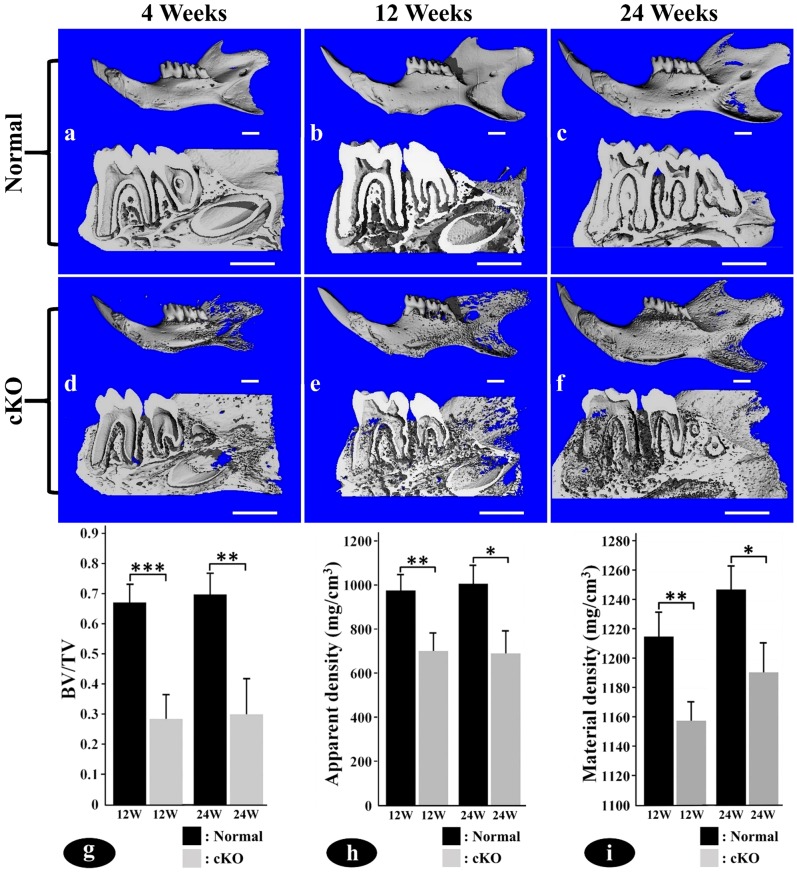
The μCT analyses of 4-, 12- and 24-week-old mice. Upper images in a–f were the μCT views of the whole mandibles, and the lower portions were the cross-section images. The normal mandibles showed even distribution of mineralized bone with smooth surfaces (upper images in a, b, c) whereas the *Fam20C*-deficient mandibles appeared porous around the molar roots (upper images in d, e, f). The porous appearance was due to the presence of excessive hypomineralized tissues or soft tissues in the alveolar bone of the cKO mice. The cross-section views showed that in all of the age groups, the alveolar bone in the furcation and interdental regions of the cKO mice (lower images in d, e, f) had significantly lower mineral density than the corresponding regions in the normal mice (lower images in a, b, c). Quantitative analysis showed that the cKO mice had significant reduction in the alveolar bone volume (g), apparent density (h) and material density (i) at 12 and 24 weeks. BV: bone volume; TV: total volume; *: P value <0.05; **: P value <0.01; ***: P value <0.001. Bar in all images equals 1.0 mm.

The μCT analyses further demonstrated the loss of alveolar bone in the cKO mice (Figure 2). At 4 weeks after birth, the alveolar bone in the furcation and interdental regions of the cKO mice (Figure 2d) showed a lower mineral density compared to the corresponding regions in the normal mice (Figure 2a). At 12 weeks, the mineral density in the alveolar bone of the normal mice (Figure 2b) was even, while the alveolar bone in the cKO mice (Figure 2e) appeared more porous, suggesting the presence of excessive osteoid or other soft tissues in the alveolar bone proper and the cortical plate. The alveolar bone abnormalities worsened as the mice aged, and the 24-week-old cKO mice (Figure 2f) showed more severe defects and porosities in the alveolar bone than did the 12-week-old mice. Quantitative analyses showed a significant reduction in the alveolar bone volume (Figure 2g), apparent density (Figure 2h) and material density (Figure 2i) in the 12- and 24-week-old cKO mice.

### Histology

**Figure 3:**
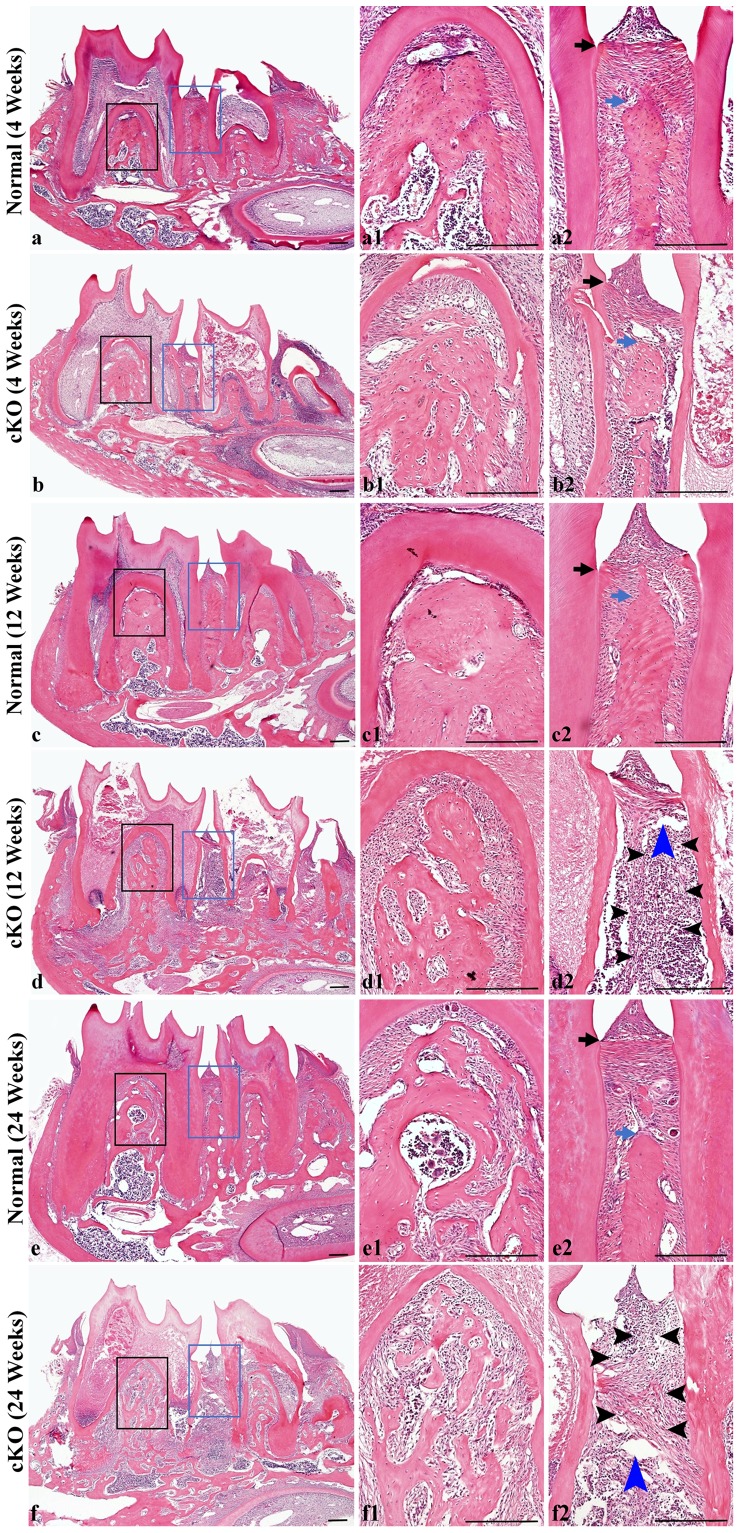
Analyses from H&E staining of periodontal tissues in 4-, 12- and 24-week-old mice. a1 and a2 were the higher magnification views of black box area and blue box area in Figure 3a (4-week-old normal mice), respectively. b1 and b2 were the higher magnification views of black and blue box area in b (4-week-old cKO mice). c1 and c2 were the higher magnification views of black and blue box area in c (12-week-old normal mice). d1 and d2 were the higher magnification views of black and blue box area in d (12-week-old cKO mice). e1 and e2 were the higher magnification views of black and blue box area in e (24-week-old normal mice). f1 and f2 were the higher magnification views of black and blue box area in f (24-week-old cKO mice). Black arrows indicate the cemento-enamel junctions. Blue arrows indicate the alveolar crests. Black arrowheads indicate the severe inflammation regions. Blue arrowheads indicate the abscesses. At 4 weeks (a, b), the height and area of alveolar bone in the interdental and interradicular regions of the cKO mice were similar to those of the normal mice, and PDL had no significant inflammation. At 12 weeks (c, d) and 24 weeks (e, f), the cKO mice showed typical features of periodontitis, which include PDL inflammation, alveolar bone loss, apical migration or destruction of junctional epithelium, and formation of periodontal pockets. Bar equals 200 µm in all images.

**Figure 4:**
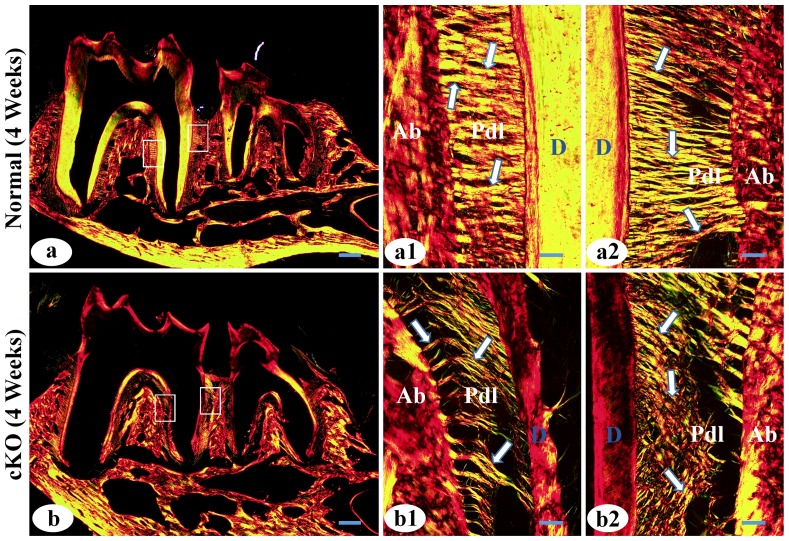
Analyses from picro-sirius red staining of periodontal tissues in 4-week-old mice. a1 and a2 were the higher magnification views of the left box area (furcation region) and right box area (interdental region) in Figure 4a (normal mice), respectively. b1 and b2 were the higher magnification views of left and right box area in b (cKO mice). Ab, alveolar bone; D, dentin; arrows indicate collagen fibers in the PDL. In the normal PDL (a, a1, a2), the thick collagen fibers were evenly distributed. In the *Fam20C*-deficient PDL (b, b1, b2), the collagen fibers were remarkably thinner and unevenly distributed, with some collagen fibers detached from the alveolar bone or root surface. Bar in a or b: 200 µm; bar in a1, a2, b1 or b2: 20 µm.

Histological evaluation of the periodontium showed bone defects, disorganization of the collagen fibers in the periodontal ligament (PDL) and detachment of the junctional epithelium, along with the formation of periodontal pockets in the 12- and 24-week-old cKO mice (Figures 3 and 4). The amounts of cellular cementum in the 12- and 24-week-old cKO mice appeared to be reduced compared to the normal mice of the same ages. The histological findings were consistent with results from the X-ray analyses, further confirming that these *Fam20C*-deficient mice developed periodontal disease.

At 4 weeks after birth, H&E staining showed that the PDL of the cKO mice did not have significant inflammation, and the junctional epithelium was at a position close to the cemento-enamel junction, similar to that observed in the normal mice (Figures 3a2, 3b2, black arrows). The height and area of the alveolar bone in the interdental and interradicular regions of the cKO mice (Figures 3b, 3b1, 3b2) were similar to those of the normal mice (Figures 3a, 3a1, 3a2). However, picro-sirius red staining showed that the collagen fibers in the PDL of the 4-week-old cKO mice were remarkably thinner and more disorganized than in the normal mice (Figure 4). Some collagen fibers in the *Fam20C*-deficient PDL appeared broken or detached from the alveolar bone or root surface (Figures 4b1, 4b2).

In the interdental area of the 12-week-old cKO mice, significant inflammation was observed in the PDL, the majority of the alveolar bone was lost, the junctional epithelium had migrated to the apical region, and deep periodontal pockets had formed (Figures 3c2, 3d2). The picro-sirius red staining revealed that the majority of the collagen fibers in the interdental area were lost (data not shown). The furcation region (Figure 3d1) in the cKO mice also showed bone loss and inflammation although the defects in this area were not as severe as those in the interdental region (Figure 3d2).

The periodontal defects in the 24-week-old cKO mice (Figures 3e, 3f) were worse than in the 12-week-old mice. At 24 weeks, nearly all of the interdental alveolar bone was lost and certain areas of the PDL were necrotized, accompanied by the formation of abscesses. Due to significant bone absorption, the alveolar bone in the furcation regions of the cKO mice became island-like (bone spicules), giving rise to a network appearance; inflammatory cells and fibroblasts were present within these networks of spicules. The picro-sirius red staining revealed that nearly all of the collagen fibers in the interdental region were broken down (data not shown).

### Backscattered and acid-etched scanning electron microscopy (SEM)

**Figure 5:**
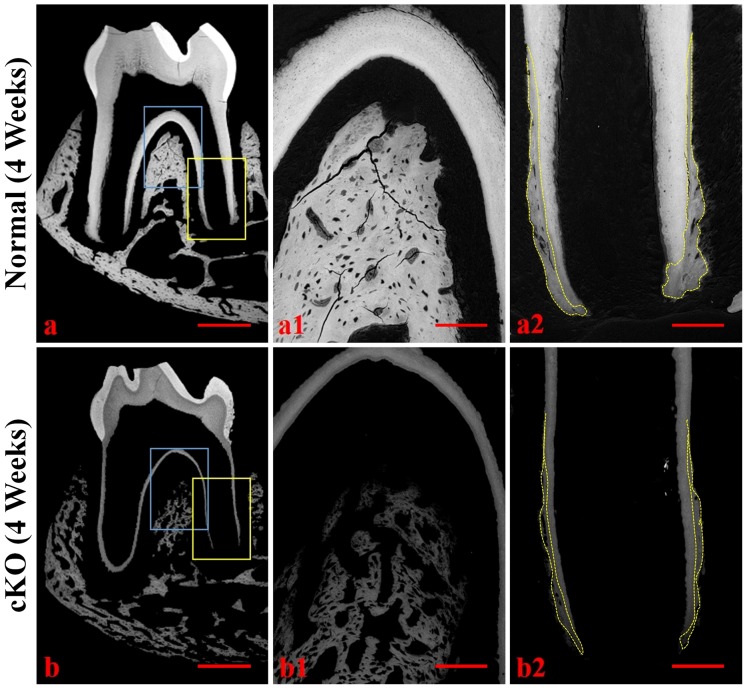
Backscattered SEM analyses of periodontal tissues in 4-week-old mice. a1 and a2 were the higher magnification views of the blue box area (furcation region) and yellow box area (apical region) in Figure 5a (normal mice), respectively. b1 and b2 were the higher magnification views of the blue box area (furcation region) and yellow box area (apical region) in b (cKO mice). In a2 and b2, the cementum was outlined by the yellow-dotted lines. In the images of backscattered SEM, the black areas represent unmineralized or hypomineralized areas, and a greater degree of whiteness represents the presence of a higher level of mineral. The network appearance in the furcation region of the cKO mice was primarily due to the presence of the unmineralized osteoid within the osseous masses; the alveolar bone images in a1 and b1 were from the upper portion of the furcation bone, which contained little or no central spongiosa. These images revealed that the alveolar bone in the furcation region of the cKO mice (b1) had a lower level of mineralization compared to the same region of the normal mice (a1). Note that the cKO mice (b2) had much less cementum than in the normal mice (a2). Bar in a or b: 500 µm; bar in a1, a2, b1 or b2: 100 µm.

Using backscattered SEM, we observed that in the 4-week-old normal mice, minerals were evenly distributed around the osteocyte lacunae in the interradicular alveolar bone of the first molar (Figures 5a, 5a1), while the mineral level was lower in the same region surrounding the osteocytes in the cKO mice (Figures 5b, 5b1). The normal mice had a considerable amount of cementum in the apical region (Figure 5a2), while the cKO mice had significantly less cementum, which also appeared to have a lower level of mineralization (Figure 5b2).

**Figure 6:**
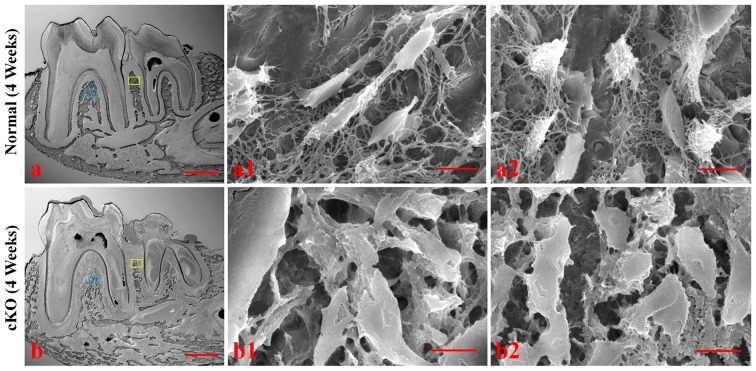
Acid-etched SEM analyses of the alveolar bone in the 4-week-old mice. a1 (normal) and b1 (cKO) were SEM images taken from the alveolar bone in the furcation region (from the blue box areas). Images of a2 (normal) and b2 (cKO) were taken from the alveolar bone in the interdental region (yellow box). The lacunae of *Fam20C*-deficient osteocytes appeared “collapsed” (b1, b2). The lacuno-canalicular networks in the cKO mice were disorganized with fewer canaliculi that appeared thicker and more randomly distributed compared to the normal mice. Bar in a or b: 500 µm; bar in a1, a2, b1 or b2: 10 µm.

The resin-infiltrated sections were acid-etched to reveal three-dimensional images of the osteocytes and their processes contained in the lacuno-canalicular systems of the alveolar bone (Figure 6). The lacunae of the normal osteocytes in the interdental region or furcation region were highly organized and regularly spaced with numerous canaliculi appearing to radiate out orderly from the osteocyte lacunae (Figures 6a1, 6a2). In comparison, the lacunae of the osteocytes in the alveolar bone of the cKO mice appeared to be larger and irregularly distributed with fewer disorganized canaliculi, giving the impression of being “collapsed” (Figures 6b1, 6b2). These observations indicated that the osteocytes and their processes in the alveolar bone were abnormal.

### Immunohistochemistry (IHC) Staining

IHC was performed to assess the presence or absence of FAM20C and to analyze the expression and distribution of BSP, OPN, DMP1, DSP, periostin and fibrillin-1 in the normal and cKO mice at 4, 12 and 24 weeks after birth. In this report, the representative images from the IHC analyses of 4-week-old mice are presented.

**Figure 7:**
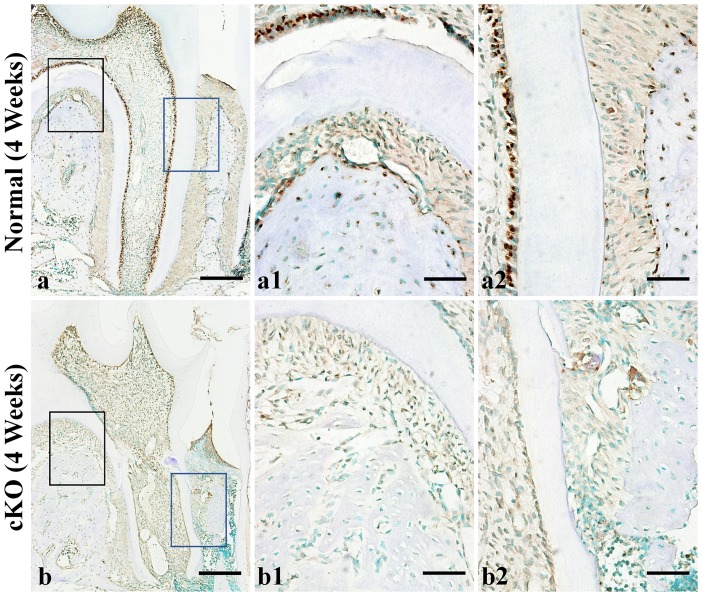
IHC analyses of FAM20C in the periodontal tissues of 4-week-old mice. The specimens were from the first molar region of mandibles in the 4-week-old normal mice (a) and cKO mice (b). a1 and a2 were the higher magnification views of the black box area (furcation region) and blue box area (interdental region) in Figure 7a (normal mice), respectively. b1 and b2 were the higher magnification views of the black and blue box area in b (cKO mice). Note the positive signal (brown color) for FAM20C in the alveolar bone and PDL of normal mice (a, a1, a2), and the lack of this molecule in the cKO mice (b, b1, b2). Bar in a or b: 200 µm; bar in a1, a2, b1 or b2: 50 µm.

Anti-FAM20C immunostaining analyses showed that in the normal mice, FAM20C was present in the odontoblasts, osteoblasts and PDL fibroblasts, while the signal for this protein was not seen in the corresponding components of the cKO mice (Figure 7). These observations indicated that in the *2.3 kb Col 1a1-Cre;Fam20C^fl/fl^* mice, FAM20C was effectively nullified in the Type I collagen-expressing cells.

**Figure 8:**
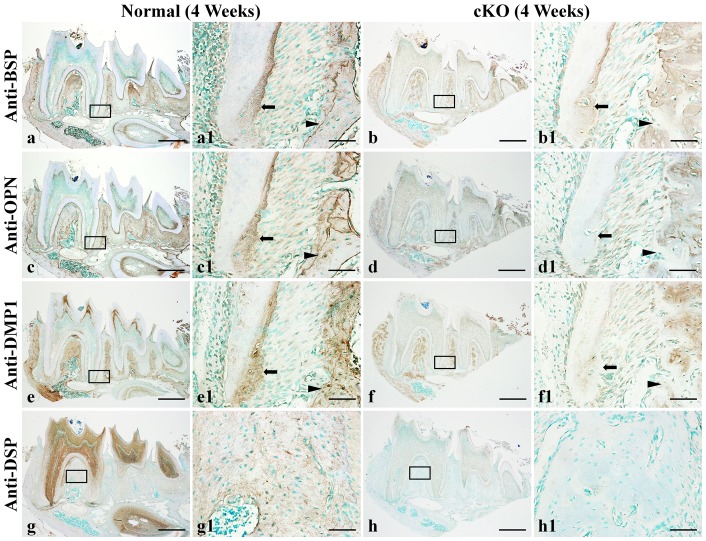
IHC analyses of SIBLING proteins in the periodontal tissues of 4-week-old mice. a1 was the higher magnification view of the box area in Figure 8a (normal mice, anti-BSP immunostaining). b1 was the higher magnification view of the box area in b (cKO mice). c1 was the higher magnification view of the box area in c (normal mice, anti-OPN). d1 was the higher magnification view of the box area in d (cKO mice). e1 was the higher magnification view of the box area in e (normal mice, anti-DMP1). f1 was the higher magnification view of the box area in f (cKO mice). g1 was the higher magnification view of the box area in g (normal mice, anti-DSP). h1 was the higher magnification view of the box area in h (cKO mice). Arrows indicate cementum, and arrow heads indicate alveolar bone. Note that the signals (brown) for BSP, OPN and DMP1 in the cementum and alveolar bone of the cKO mice were weaker compared to the same tissues of the normal mice. DSP signals were clearly observed in the alveolar bone of normal mice, but were undetectable in the same tissue of the cKO mice. These data indicate that the levels of these SIBLING family members were reduced in the periodontal tissues of the cKO mice. In the IHC analyses for each type of the antibodies, the specimens from the normal and cKO mice from the same litters were stained in the same batch of experiments. Bar in a, b, c, d, e, f, g or h: 500 µm; bar in a1, b1, c1 d1, e1, f1, g1 or h1: 50 µm.

In the 4-week-old normal mice, BSP was mainly detected in the alveolar bone and cementum, and the immunoreactivity was stronger along the reversal lines in the alveolar bone (Figures 8a, 8a1). The signal for BSP was weaker in the cementum (arrows) and alveolar bone (arrowheads) of the cKO mice (Figures 8b, 8b1) compared to the two tissues of the normal mice (Figures 8a, 8a1). Additionally, BSP in the *Fam20C*-deficient alveolar bone showed a diffused distribution pattern (Figure 8b1), in contrast to the protein of the normal mice that was concentrated along the reversal lines in the alveolar bone (Figure 8a1).

In the normal mice, OPN was detected in the alveolar bone, cementum and PDL (Figures 8c, 8c1). In the cementum and alveolar bone of the cKO mice (Figures 8d, 8d1), the level of OPN was remarkably reduced in comparison to their normal littermates. The level of OPN in the PDL of cKO did not seem to be significantly different from that of the normal mice.

In the periodontium of the normal mice, DMP1 was observed in the alveolar bone and cementum (Figures 8e, 8e1). The level of DMP1 was remarkably lower in the *Fam20C*-deficient alveolar bone and cementum (Figures 8f, f1) than in the normal tissues.

In the periodontium of normal mice, DSP was mainly detected in the alveolar bone, in particular, the alveolar bone of the furcation region (Figures 8g, 8g1). DSP was undetectable in the alveolar bone of the cKO mice (Figures 8h, 8h1).

**Figure 9:**
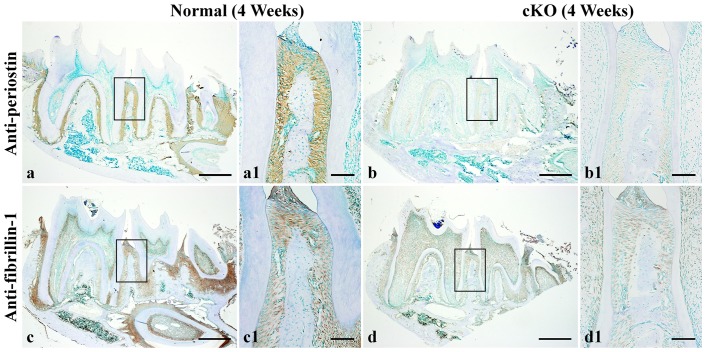
IHC analyses of periostin and fibrillin-1 in the periodontal tissues of 4-week-old mice. a1 was the higher magnification view of the box area in Figure 9a (normal mice, anti-periostin). b1 was the higher magnification view of the box area in b (cKO mice). c1 was the higher magnification view of the box area in c (normal mice, anti-fibrillin-1). d1 was the higher magnification view of the box area in d (cKO mice). Strong signals for periostin were seen in the PDL, in particular, along the collagen fibers in the normal mice (a, a1). The level of periostin in the PDL of the cKO mice was reduced (b, b1). Fibrillin-1 signals were strong in certain areas of the PDL and its signals were weaker in the PDL of cKO mice (d, d1) compared to the normal mice (c, c1). Bar in a, b, c or d: 500 µm; bar in a1, b1, c1 or d1: 100 µm.

In the normal mice, strong signals for periostin were observed across the PDL, with an accentuated accumulation along the thick collagen fibers (Figures 9a, 9a1). The level of periostin in the PDL of the cKO mice was dramatically reduced (Figures 9b, 9b1).

Strong signals for fibrillin-1 were detected in certain areas of the PDL of the normal mice (Figures 9c, 9c1). The fibrillin-1 signals were weaker in the PDL of the cKO mice (Figures 9d, d1) than in the normal mice.

## Discussion

FAM20C has been studied only to a limited extent. Previously, we analyzed the spatiotemporal expression of FAM20C in mouse tissues and found that this protein is expressed at significant levels in osteoblasts, cementoblasts and PDL fibroblasts [9]. In this study, we analyzed the periodontal tissues of the *2.3 kb Col 1a1-Cre;Fam20C^fl/fl^* (cKO) mice, in which *Fam20C* was inactivated in the cells expressing Type I collagen. Since osteoblasts, cementoblasts and PDL fibroblasts express Type I collagen, the alveolar bone, cementum and PDL in the cKO mice were *Fam20C*-deficient, allowing us to analyze the effects of *Fam20C* inactivation on the health of periodontium.

At 4 weeks after birth, histology analyses using H&E staining revealed that there was no obvious inflammation in the PDL and no significant migration of the junctional epithelium in the *Fam20C*-deficient mice. However, picro-sirius red staining showed that the collagen fibers in the *Fam20C*-deficient PDL were very thin, sparsely distributed and disorganized. The backscattered SEM analyses showed that the mineralization level of the alveolar bone and cementum in the cKO mice was lower than in the normal mice, and the cKO mice also had less cementum. The acid-etched SEM analyses demonstrated that the lacunae of osteocytes in the *Fam20C*-deficient alveolar bone appeared to have “collapsed”, and the process-encompassing canaliculi were disorganized. There was a sharp reduction of the SIBLING proteins: BSP, OPN, DMP1 and DSP in the *Fam20C*-deficient alveolar bone and/or cementum. Previous studies have shown that loss-of-function of BSP [18], DMP1 [16], or DSPP [19] leads to periodontal defects in mice. The reduction of these SIBLING molecules in the periodontium of the cKO mice could be a contributing factor to the development of periodontal disease in these mice at later stages. Periostin is an adhesion molecule produced by the fibroblasts and secreted into the PDL [21]. Studies have shown that inactivation of periostin leads to periodontal disease in mice [24], [25], [28]. In the present investigation, we showed a remarkable reduction of periostin in the PDL of the cKO mice. Another ECM molecule, fibrillin-1, whose inactivating mutations are associated with severe periodontal diseases [26], [27], was also reduced in the PDL of the cKO mice. These structural and molecular changes in the cKO mice indicate the *Fam20C*-deficient periodontium had intrinsic (inherent) defects. Collectively, these intrinsic defects may lead to the severe periodontal disease observed in the 12- and 24-week-old cKO mice.

At 12 or 24 weeks after birth, the cKO mice revealed a significant reduction of alveolar bone and cementum, remarkable inflammation in the PDL, formation of deep periodontal pockets, and disorganization of PDL fibers. These findings demonstrated clearly that the *Fam20C*-deficient mice developed periodontal disease. It should be noted that mice younger than 12 months do not naturally develop periodontal diseases [37], and thus, the periodontal disease in the cKO mice must be attributed to the inactivation of *Fam20C*. The cKO mice also had inflammation in the dental pulp at 12 or 24 weeks, which might spread to the PDL via the apical foremen. Therefore, the inflammation in the PDL at these stages may be attributed to two factors: 1) direct infiltration of bacteria from the periodontal pockets that were formed in association with the intrinsic defects of *Fam20C*-deficient periodontium (primary), and 2) spreading of inflammation from the infected pulp (secondary). We believe that the inherent defects caused the lack of proper formation of alveolar bone, cementum and PDL, which subsequently leads to the apical migration of the epithelial attachment, inflammation in the PDL and formation of periodontal pockets, while the secondary effects (spreading of inflammation from the infected pulp) might further aggravate the periodontal disease in the cKO mice. These observations indicate that FAM20C plays a fundamental role in maintaining the structural integrity of the periodontal structures. While the 4-week-old cKO mice had intrinsic defects in their periodontium, they did not form periodontal pockets. The 12-week-old cKO mice formed deep periodontal pockets and the defects became much worse at 24 weeks after birth. These observations indicate that the periodontal deterioration progressed rapidly in the absence of FAM20C.

*In vitro* studies have revealed that FAM20C is a Golgi kinase that phosphorylates serine residues in the S-X-E motifs of members in the secretory calcium binding phosphoprotein family [12], [13], which includes the SIBLING molecules and certain enamel proteins [13], [38]. Mouse periostin has three S-X-E motifs in its amino acid sequence [30], [31], and mouse fibrillin-1 has seven S-X-E motifs [29]; thus, these two ECM molecules are potential substrates of FAM20C. In this investigation, we observed a significant reduction of the SIBLING proteins (BSP, OPN, DMP1, DSP), periostin and fibrillin-1. At this point, we do not have a clear answer to the question of why the inactivation of *Fam20C* leads to the reduction of these secretory proteins. We hypothesize that a partial or complete failure of the phosphorylation of these ECM proteins may send feedback signals to the corresponding cells in the cKO mice and instruct the cells to reduce the synthesis of these proteins in order to avoid “wasting” their products. It is also possible that the ECM proteins with a partial or complete failure in phosphorylation may be degraded faster than their natural forms, leading to the reduction of these molecules in the ECM of the periodontal tissues in the mutant mice. Clearly, future studies are warranted to examine the phosphorylation status of these ECM proteins in the *Fam20C*-deficient tissues.
